# Visual reinforcement audiometry and its association with neonatal hearing screening and the offer of speech therapists, services and equipment

**DOI:** 10.1590/2317-1782/e20240237en

**Published:** 2026-01-30

**Authors:** Monique Ramos Paschoal Dutra, Rodrigo Oliveira da Fonsêca, Aryelly Dayane da Silva Nunes-Araújo, Sheila Andreoli Balen

**Affiliations:** 1 Maternidade Escola Januário Cicco – MEJC, Empresa Brasileira de Serviços Hospitalares – EBSERH - Natal (RN), Brasil.; 2 Laboratório de Inovação Tecnológica em Saúde – LAIS, Universidade Federal do Rio Grande do Norte – UFRN - Natal (RN), Brasil.; 3 Departamento de Fonoaudiologia, Universidade Federal do Rio Grande do Norte – UFRN - Natal (RN), Brasil.

**Keywords:** Hearing, Hearing Loss, Public Health Policy, Unified Health System, Health Information Systems

## Abstract

**Purpose:**

To analyze the outpatient production of the visual reinforcement audiometry (VRA) in the Unified Health System (SUS), in the Federation Units (UFs) of Brazil, between 2004 and 2021, and its association with Neonatal Hearing Screening (NHS) and offering audiologists, hearing health services and VRA equipment.

**Methods:**

Ecological mixed study, which used data from the SUS Outpatient Information System, of the National Registry of Health Establishments and of the Brazilian Institute of Geography and Statistics. The dependent variable was the rate of performance of the “visual reinforcement audiometry (air/bone route)” procedure, per 10 thousand inhabitants, while the independent variables were the rates of NHS, speech therapists, hearing health services and VRA equipment. Descriptive and inferential statistics and spatial analysis were performed.

**Results:**

The VRA rate in Brazil was 11.8/10 thousand inhabitants between 2004 and 2021. In the UFs, VRA rates varied from 0 to 125.1/10 thousand inhabitants in the periods studied. The lowest rate for this exam was recorded in the 2004-2006 triennium, while the highest was seen in the 2007-2009 triennium. It was found that there was no significant correlation between the VRA performance rate for the 2016-2018 three-year period with the rates of NHS, speech therapists, hearing health services and VRA equipment.

**Conclusion:**

The realization of the VRA is restricted and discrepant among Brazilian states and does not show any association with the variables investigated, indicating the need for measures to improve access to this exam in the SUS.

## INTRODUCTION

The World Health Organization estimates that by 2050, around 2.5 billion people will have some degree of hearing loss^(1)^. Hearing loss can cause substantial damage in childhood by compromising language development, communication, learning, and sociability, which require timely diagnosis and intervention^(1,2)^, as recommended^(3)^.

Identifying a child's need for hearing intervention must occur within the first six months of life^(2)^, and is guaranteed in Brazil by the National Health System (SUS), which provides the population with access to different health services^(4)^. Historically, the SUS has been marked by advances that have promoted improvements in the health of Brazilians^(5)^. In hearing health care, one of the milestones was the establishment of the National Hearing Health Care Policy (PNASA) by Ordinance GM/MS No. 2,073 of 2004, aimed at expanding coverage for people with hearing impairments through a coordinated and integrated care network across all levels of the SUS^(6)^. In 2010, another essential advance was the publication of Federal Law No. 12,303, which made it mandatory to perform Evoked Otoacoustic Emissions (EOAE) testing on all children born in hospitals and maternity wards^(7)^.

PNASA was revoked in 2011, shortly after the establishment of the National Plan for the Rights of Persons with Disabilities – Living Without Limits Plan, through Decree No. 7,612, which coordinated policies, programs, and actions aimed at improving access to education, health care, social inclusion, and accessibility in the country^(8)^. The following year, the Care Network for People with Disabilities (RCPD) was created by Ordinance GM/MS No. 793 to expand access and improve the quality of care for people with disabilities^(9)^.

In parallel with advances in Brazilian legislation on hearing health care, it is well known that, for effective diagnosis and intervention, speech therapists and specialized equipment are essential, in light of protocols based on scientific evidence, to determine the type and degree of hearing loss and subsequently guide the necessary intervention^(3)^. From this perspective, pediatric audiological diagnosis has been improved in identifying hearing loss in children^(10)^, using the cross-check principle^(11)^, which consists of a battery of tests to confirm and characterize the type, degree, and configuration of hearing loss. Thus, recommendations in pediatric audiology support that behavioral, electrophysiological, and electroacoustic assessments should be incorporated into a comprehensive evaluation to confirm results across multiple procedures^(12)^.

Audiological diagnosis performed at Specialized Rehabilitation Centers or Hearing Health Services in Brazil must follow scientific evidence-based recommendations. In this sense, between birth and 5-6 months of age, audiological diagnosis prioritizes electrophysiological and electroacoustic procedures, along with observations of auditory behavior, given the child's neuromaturational processes during this period. Behavioral or psychoacoustic assessment of hearing is considered the gold standard for estimating minimum hearing levels; therefore, for children aged 5/6 to 24 months, the recommended procedure is visual reinforcement audiometry (VRA)^(3)^.

The VRA is based on a conditioned response (turning the head horizontally) to pure-tone and speech stimuli, with visual stimuli reinforcing the response^(13)^. When VRA is conducted according to careful stimulus, response, and conditioning protocols, valid and reliable results can be obtained^(3)^, which correlate with hearing thresholds obtained later in play audiometry^(14)^. Furthermore, the VRA results suggest a strong correlation between minimum response levels in the VRA and electrophysiological thresholds, as measured by Specific Frequency Brainstem Auditory Evoked Potential tests^(10,15)^.

The Guidelines for Neonatal Hearing Screening (NHS) in Brazil recommend audiological assessment using VRA testing with insert earphones and acoustic immittance measurements during the audiological diagnosis (failure in NHS) and hearing monitoring (passes NHS with Hearing Loss Risk Indicator) stages^(16)^. In addition, the VRA is indicated for the assessment of children over 24 months of age who show developmental delays or restricted interests, such as in Autism Spectrum Disorder ^(13)^.

Over the years, studies have investigated the decline in VRA registrations in Brazil. It is also worth noting that knowledge of the evolution of this exam, both interregional and intraregional, remains limited^(17,18)^. Added to this is the scenario of profound discrepancies in hearing health care that still exist in the country^(19,20)^, such as the unequal distribution of hearing health services^(17)^.

On another level, it should be recognized that there has been a significant increase in the supply of speech therapists in the SUS^(21)^ and NHS equipment^(19)^. Given these issues, it is necessary to understand the implementation of VRA in relation to its interface with these factors, to support health decision-making, optimize public resources, and ensure consistency with scientific evidence. The British Society of Audiology recently published updated recommendations on best practices for performing VRA in pediatric audiological diagnosis^(22)^.

In view of the above, the objective of this study was to analyze the outpatient production of VRA in the SUS, in the Federative Units (UFs) of Brazil, between 2004 and 2021, and its association with NHS and the supply of speech therapists, hearing health services, and VRA equipment.

## METHOD

This is a mixed-methods ecological study, with units of analysis the Brazilian states. The sources used for data collection were the SUS Outpatient Information System (SIA/SUS), the National Register of Health Establishments (CNES), and the Brazilian Institute of Geography and Statistics (IBGE). Data collection took place in July 2022.

The time frame investigated covered the period from January 2004, given the implementation of PNASA in that year, to December 2021. For analysis purposes, the period was divided into six intervals, totaling 18 years, namely: 2004-2006; 2007-2009; 2010-2012; 2013-2015; 2016-2018; and 2019-2021, referring to rates 1, 2, 3, 4, 5, and 6, respectively.

Access to the data was obtained on the platform of the SUS IT Department – DATASUS, of the Ministry of Health, through the items “health information (TABNET)”, “health care”, and “place of care.” Subsequently, the tests “visual reinforcement audiometry (air/bone conduction)” and “evoked otoacoustic emissions for hearing screening” were selected. The procedure chosen to represent NHS was the EOAE examination, as it is the most widely used in scientific literature. The extraction of data related to the number of speech therapists was sequenced by “care network,” “CNES – human resources,” and “professionals”.

In DATASUS, the quantities of hearing health services and VRA equipment in the SUS were located by considering the states and the period “December of each year”. For hearing health services, “reports – specialized services – hearing health care service” was selected, and in the “facility search” field, “specialized rehabilitation center” was specified. As for VRA equipment, data was collected by accessing “reports – equipment” and “type of equipment – audiology equipment – complete visual reinforcement system (VRA)”. The population estimates used were derived from IBGE, which provides annual projections by state. This study used a constant of 10,000 for the population between 0 and 4 years of age to calculate the rates.

The dependent variable was described by the rate of performance of the “visual reinforcement audiometry (air/bone conduction)” procedure per 10,000 inhabitants between 0 and 4 years of age, while the independent variables were the rates of NHS, speech therapists, hearing health services, and VRA equipment, collected only for the penultimate three-year period, since the COVID-19 pandemic occurred in the last three years. For all variables, rates were calculated by averaging the variable's quantity over the three years and the estimated population for the second year, as illustrated in Chart 1.

**Chart 1:** Presentation of the study variables with the definition and formula of the calculations used

Variable	Formula
Visual Reinforcement Audiometry completion rate	Average number of Visual Reinforcement Audiometry procedures in the three years/population aged 0 to 4 years in the second year of the three years per 10,000 inhabitants.
Neonatal Hearing Screening Implementation Rate	Average number of “Evoked Otoacoustic Emissions for Hearing Screening” procedures in the three years/population aged 0 to 4 years in the second year of the three years per 10,000 inhabitants.
Speech therapists' rate	Average number of speech therapists working in the Unified Health System in the three years/population, aged 0 to 4 years, in the second year of the three years, per 10,000 inhabitants.
Hearing health services rate	Average number of hearing health services and specialized rehabilitation centers in the Unified Health System in the three years/population, aged 0 to 4 years, in the second year of the three years, per 10,000 inhabitants.
Visual Reinforcement Audiometry Equipment Rate	Average number of visual reinforcement audiometry devices available in the Brazilian National Health System in the three years/population aged 0 to 4 years, in the second year of the three years, per 10,000 inhabitants.

The data were compiled and organized in a database using the Statistical Package for the Social Sciences, version 18.0, in which descriptive and inferential statistics were performed. Initially, the normality of the distribution of the dependent variable was tested using the Shapiro-Wilk test, with α = 5%. Since the distribution was not standard, measures of central tendency and dispersion, along with Spearman's test, were employed to examine the correlations between the dependent and independent variables, with α = 5%. Next, using TabWin software, version 4.15, an exploratory analysis was performed of the rates of performance of the “visual reinforcement audiometry (air/bone conduction)” procedure per 10,000 inhabitants aged between 0 and 4 years in the six periods examined, using quartile distribution. On the maps, the intensity of the gray levels is related to the magnitude of the rates, i.e., the darker the color, the higher the values.

This study was exempted from review by the Research Ethics Committee due to its ecological design and the collection of data from a public domain database, in accordance with Resolution No. 510/2016 of the National Health Council^(23)^.

## RESULTS

**Figure 1:**
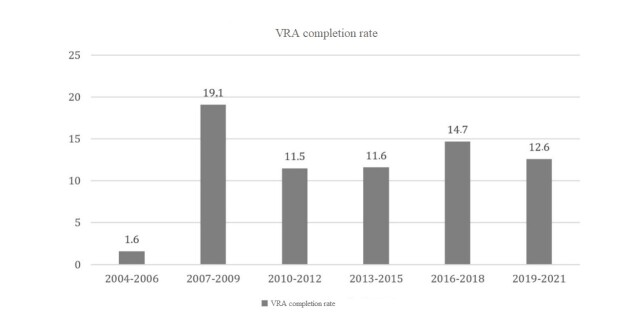
VRA completion rate per 10,000 inhabitants, aged between 0 and 4 years old, from 2004 to 2021 in Brazil

During the period investigated, the rate of VRA in Brazil was found to fluctuate between 1.6 and 19.1 per 10,000 inhabitants among children aged 0 to 4. The lowest rate was recorded between 2004 and 2006, and the highest in the following period, from 2007 to 2009 (Figure 1).

**Caption:** Taxa de realização de VRA = VRA completion rate

**Figure 2:**
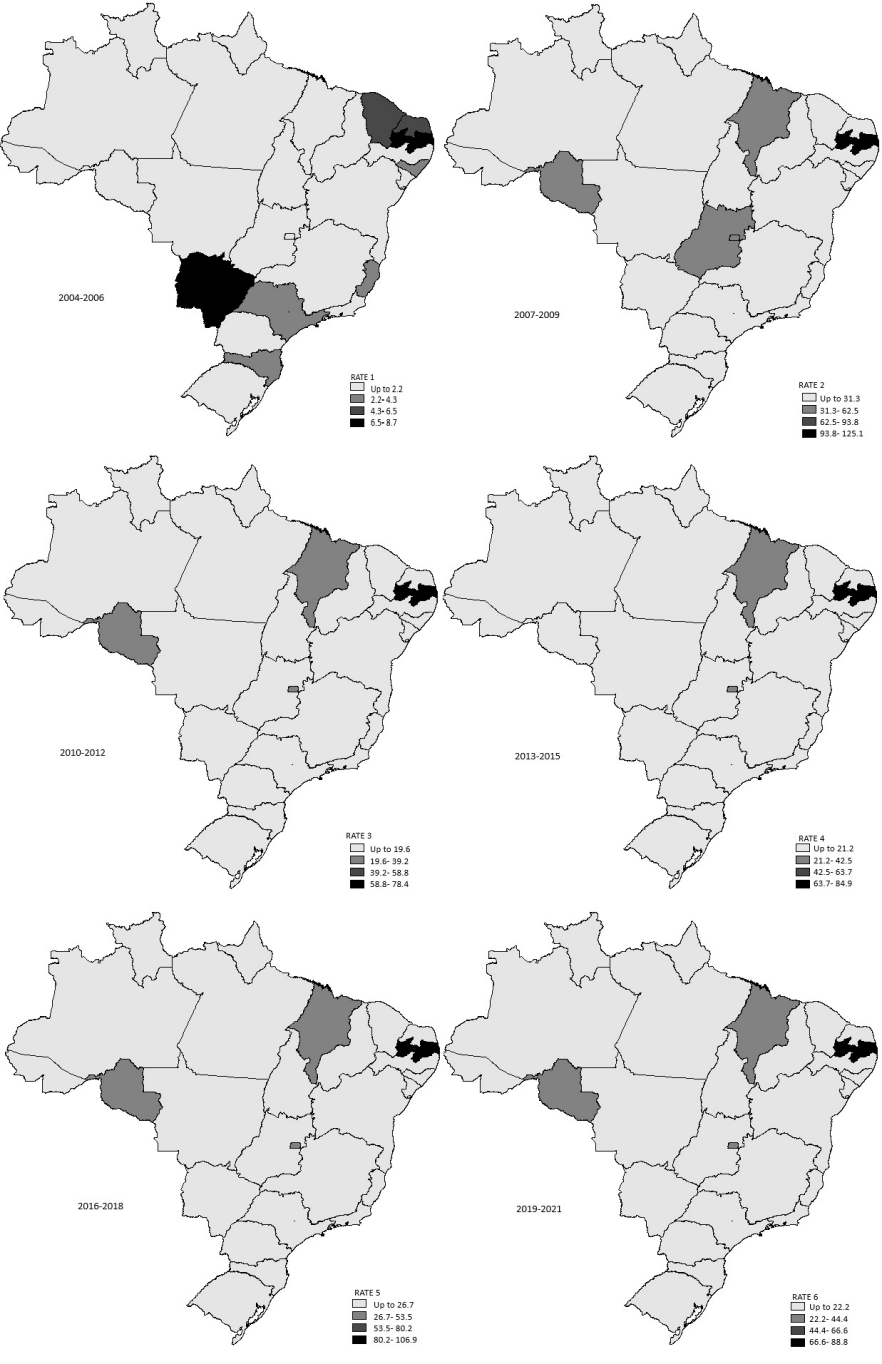
Spatial distribution of the VRA completion rate for every 10,000 inhabitants between 0 and 4 years of age, from 2004 to 2021, by Brazilian state

The Northeast region stood out, as Paraíba had the best rates on the maps for all three-year periods analyzed, and Maranhão was among the states with the highest rates on the map for the 2007-2009 period. The units in Rondônia and the Federal District were also highlighted. The map for the 2004-2006 triennium showed the lowest rates, ranging from 0 to 8.7 per 10,000 inhabitants aged 0-4 years, with greater variation between units, while the map for the 2007-2009 triennium showed the highest rates, ranging from 31.3 to 125.1 (Figure 2).

According to Table 1, Brazil's NHS rate for 0- to 4-year-olds was 1,483.20/10,000 inhabitants, ranging from 581.3 in Mato Grosso to 3,324.8 in Amapá (mean = 1,729.6; SD = 1,387.8-2,071.4). Regarding the rate of speech therapists, Brazil's rate was 13.70/10,000 inhabitants aged 0-4 years, with Minas Gerais having the highest rate, 18.90, and Amazonas having the lowest, 4.4 (mean = 11.3; SD = 9.7-12.9). As for hearing health services, the rate in Brazil was 0.4/10,000 inhabitants aged 0-4 years, ranging from 0.1 in Alagoas to 1.2 in Paraná (Md = 0.3; IIQ = 0.2). Finally, the rate of VRA equipment in Brazil was 0.13/10,000 inhabitants between 0 and 4 years of age, with the highest rate recorded in Goiás, at 0.6, and the units in Rondônia, Acre, Amapá, Tocantins, Maranhão, and Sergipe had rates with zero values (Md = 0.1; IIQ = 0.2). Table 1 considered the IBGE's standardized sequence for ordering the states, beginning with Rondônia and ending with the Federal District.

**Table 1:** Rates of hearing screening, NHS, speech therapists, hearing health services, and hearing screening equipment per 10,000 inhabitants between the ages of 0 and 4, from 2016 to 2018, by Brazilian state

FU	TXVRA	TXTAN	TXFONO	TXSS	TXEQUIP
Rondônia	26.80	891.40	9.40	0.4	0.00
Acre	0.00	2,018.60	5.00	0.4	0.00
Amazonas	0.20	1,216.30	4.40	0.2	0.10
Roraima	0.00	2,746.80	7.50	0.2	0.10
Pará	16.20	1,089.70	5.60	0.3	0.10
Amapá	0.00	3,324.80	8.80	0.5	0.00
Tocantins	0.30	1,451.90	9.40	0.8	0.00
Maranhão	39.90	786.50	6.50	0.3	0.00
Piauí	0.10	2,530.90	12.90	0.2	0.20
Ceará	9.30	1,217.70	9.70	0.2	0.10
Rio Grande do Norte	22.80	1,611.30	13.10	0.6	0.40
Paraíba	106.90	2,558.60	13.30	0.3	0.10
Pernambuco	0.70	827.20	10.70	0.4	0.10
Alagoas	4.10	1,263.10	9.30	0.1	0.10
Sergipe	0.00	1,630.00	9.50	0.3	0.00
Bahia	17.80	1,003.90	8.20	0.3	0.10
Minas Gerais	4.00	1,261.30	18.90	0.3	0.10
Espírito Santo	8.30	1,417.00	13.00	0.3	0.20
Rio de Janeiro	5.60	1,036.30	17.20	0.4	0.10
São Paulo	21.50	1,167.90	18.70	0.5	0.20
Paraná	12.70	3,306.90	16.90	1.2	0.20
Santa Catarina	1.90	2,010.30	17.20	0.6	0.20
Rio Grande do Sul	6.50	3,259.50	15.20	0.6	0.10
Mato Grosso do Sul	12.50	3,055.60	13.30	0.5	0.30
Mato Grosso	2.80	581.30	8.80	0.2	0.20
Goiás	23.20	963.80	12.10	0.5	0.60
Distrito Federal	30.70	2,471.70	12.20	0.3	0.00
Total	14.70	1,483.20	13.70	0.4	0.13

**Caption:** FU = Federal Unit; TXVRA= VRA completion rate; TXTAN = Neonatal Hearing Screening rate; TXFONO = speech therapist rate; TXSS = Hearing health services rate; TXEQUIP = VRA equipment rate

In the inferential statistical analysis, no significant correlation was observed between the rate of VRA performance in the penultimate three-year period and the rates of NHS, speech therapists, hearing health services, and VRA equipment. The rate of speech therapists showed a statistically significant correlation with the rates of hearing health services and VRA equipment (Table 2).

**Table 2:** Analysis of the correlation between VRA rates, NHS, speech therapists, VRA equipment, and hearing health services per 10,000 inhabitants between the ages of 0 and 4, from 2016 to 2018, in Brazil

Variables	TX VRA	TX TAN	TX FONO	TX EQUIP	TX SS
TX VRA	1.000				
TX TAN	-0.269	1.000			
TX FONO	0.241	0.257	1.000		
TX EQUIP	0.191	0.015	0.486^*^	1.000	
TX SS	0.138	0.274	0.464*	0.369	1.000

**Caption:** TXVRA = VRA completion rate; TXTAN = Neonatal Hearing Screening rate; TXFONO = speech therapist rate; TXEQUIP = VRA equipment rate; TXSS = Hearing health services rate

* moderate correlation

## DISCUSSION

The results of this study allowed us to identify the variability in the performance of VRA in the SUS over the years. The states analyzed simultaneously exhibited significant disparities in terms of NHS rates, speech therapists, hearing health services, and VRA equipment. This context is considered worrying, given that this examination, which is essential for audiological diagnosis in children aged 5/6 to 24 months, has undergone significant temporary declines.

The highest rates of VRA implementation in Brazil, reported between 2007 and 2009, are in line with the progress that PNASA has made in hearing health care^(24,25)^, so much so that, with its establishment, access to care in this area has been progressively expanded, reflecting an increase in the performance of audiological procedures^(17)^. Under this premise, the lower rates of VRA implementation recorded between 2004 and 2006 are linked to the still recent process of implementing PNASA in the country, which allows us to understand these findings. It should also be noted that PNASA did not present specific recommendations for performing VRA in the SUS^(6)^.

Given the overall trend observed in this study, it is worth highlighting the behavior of VRA rates in the SUS in the other three-year periods analyzed, since from 2010 to 2012, there was a sharp decrease when compared to the peak recorded between 2007 and 2009, followed by a slight positive variation from 2013 to 2015 and declines in the subsequent three-year periods. The results corroborate the study conducted in Salvador, Bahia, which pointed to a general upward trend in outpatient hearing health care, peaking in 2007, with variations in the other years surveyed^(26)^. The investigated scenario corroborates a study that found an increase in NHS coverage rates in Brazil between 2008 and 2015^(27)^.

In view of this, it is worth considering changes to policies related to hearing health in the SUS, such as the revocation of PNASA, the publication of Brazilian Federal Law No. 12,303, the implementation of the Living Without Limits Plan, and the creation of the RCPD. Amid the transition to such measures, there was an increase in the number of audiological procedures performed in the SUS, albeit unevenly across Brazilian regions and procedure types, such as a 28.26% decrease in VRA procedures^(18)^.

According to the VRA findings in the SUS for the three-year periods analyzed, the highest rates were observed in Paraíba, Maranhão, Rondônia, and the Federal District. Paraíba, which appeared on the maps for all three-year periods with the most prominent rates for the procedure, has already emerged in the literature due to its prominence in terms of the best coverage of NHS in Brazil^(27)^ and speech therapists in the SUS^(19)^. Contrary to these results, the behavior of NHS rates and speech therapists in the SUS from 2016 to 2019 was different, with greater amplitudes in Amapá and Minas Gerais, respectively.

The higher NHS rates compared with VRA rates stem from public policies and the need to perform the procedure on all live births in Brazil^(16)^. The negative correlation between the VRA completion rate and the NHS rate reveals a gap in the Universal NHS program, as locations where screening is most widely performed conduct fewer VRA tests, suggesting evasion at the monitoring and diagnosis stages^(28)^. On the other hand, it is worth noting that the health information system used does not specify the age at which the audiological diagnosis was completed, which, in turn, is prone to variability due to various aspects inherent to Brazilian states.

The distribution of speech therapists in the SUS in the South and Southeast regions is consistent with the history of undergraduate courses in Speech Therapy in Brazil and with social indicators^(21)^, given that the highest rates of speech-language pathologists were found in states in these regions, where there is a predominance of audiology specialists and training programs in the field, which are indispensable elements in the effectiveness of the care provided^(24)^. Therefore, the implementation of VRA depends on audiologists' experience and specific training during its execution, based on scientific evidence^(13,22)^. It is worth noting that speech therapists have other areas of expertise within the SUS, in addition to audiology, and their work is increasingly necessary within multidisciplinary teams at all levels of complexity.

Additionally, one potential challenge in carrying out this procedure is the availability of resources in hearing health services^(29)^. Within this perspective and based on the findings of this study, the states demonstrated limited availability of VRA equipment, with some states, especially in the North and Northeast regions, reporting zero rates. These findings reveal the importance of investing in physical resources in the SUS^(4,19)^, given that the equipment needed to perform the VRA involves a specific system with a larger cabin and particular materials, such as light-up toys^(22)^.

It is well known that speech therapy services require public investment for their maintenance, especially in audiology, which involves more costly procedures. In this regard, scholars examining public spending on outpatient speech therapy services concluded that audiology accounted for 95.4% of the resources allocated to these services, with gradual growth in investment until 2011 and instability thereafter. Among the regions, the most significant shares of spending were recorded in the Southeast, Northeast, South, Midwest, and North, respectively, with notable inequalities^(25)^.

By reporting on regional inequalities, this study fits within the context of striking discrepancies in Brazil regarding the effectiveness of healthcare and access to services^(4)^. Although regional disparities are significant, they have been mitigated by measures taken by the SUS to address this issue^(5)^. In terms of hearing healthcare, between 2008 and 2012, there was an expansion in the accreditation of new hearing healthcare services, especially in the North, Northeast, and Central-West regions, as well as in audiological procedures in the SUS, which have a direct impact on outpatient care^(30)^.

Nevertheless, according to the results of this study, inequalities in hearing health service rates were observed, with lower rates in states in the North and Northeast regions and higher rates in states in the South region, where, in 2010, estimated service coverage had already become the highest in the country^(17)^. It is therefore understandable that gaps in the accreditation of these services are likely to exacerbate existing regional inequalities further, affecting users' access to hearing healthcare.

The findings of this study can be explained by the lack of training for speech therapists in the field of pediatric audiology, which highlights the need for greater incorporation of this topic into academic training, and by the fact that there is a concentration of other audiological procedures^(17,18)^, opening space for debates involving more effective strategies to improve the stages of diagnosis^(28)^. In addition, it should be noted that VRA requires the presence of two evaluators, unlike other audiological procedures, and a specific booth with larger dimensions^(22)^. At the heart of these specificities, it is worth noting that, given technological advances and the increased acquisition of equipment for more objective testing, the performance of VRA in the SUS has become less frequent^(17)^.

The limitations of this study include reliance on data from health information systems, which may contain missing or underreported data, and the lack of mechanisms to measure these issues. In addition, the data included for inhabitants aged 0-4 years, due to the quality of the sources used, also represents a limitation of health information systems. There are no references regarding VRA rates, as there are no national studies with prevalence figures for hearing impairment in children, which limits in-depth analysis of both the rates and NHS failure rates.

The non-inclusion of the Brainstem Auditory Evoked Potential as a NHS exam and the rate calculations, which did not exclude the population with access to supplemental care, can also be mentioned as limitations. The use of the state as the unit of analysis presents values that may differ depending on the unit of analysis used, taking into account the particularities that exist between municipalities.

Despite this, the use of data from health information systems helps to recognize the reality of hearing health in the SUS and identify the states that need greater attention, contributing to assessments for the planning, implementation, and improvement of public health services and actions.

Given the importance of timely and accurate audiological diagnosis for children, scientific organizations have recommended, through indicators, the evaluation of health services and quality monitoring^(3,16,22)^. For Brazil, it is suggested that aspects related to the acquisition and maintenance of human, physical, and material resources be included in public policies, as well as studies analyzing audiological procedures aimed at intervening in hearing impairment, such as individual sound amplification devices and cochlear implants.

## CONCLUSION

The performance of VRA has shown significant declines over the years in Brazil, along with discrepancies in its distribution among states, pointing to the need for measures to minimize the impact of the restriction on this test in the SUS. The lack of correlation between VRA and NHS rates reflects the discontinuity of NHS programs: states with high NHS coverage do not show higher VRA testing rates, and states with more speech therapists, hearing health services, and VRA equipment perform fewer tests.

Given these findings, it can be inferred that disparities in VRA performance in the SUS negatively impact hearing health care, to the extent that young children may be denied access to this test during the audiological evaluation process.
