# Supplementary material for: Visual reinforcement audiometry and its association with neonatal hearing screening and the offer of speech therapists, services and equipment
Source: Codas. 2026 Jan 30;38(1):e20240237. doi: 10.1590/2317-1782/e20240237en (PMC12978325; doi:10.1590/2317-1782/e20240237en)
Supplement: Translation

# Translation: Audiometria de reforço visual e sua associação com a triagem auditiva neonatal e a oferta de fonoaudiólogos, serviços e equipamentos

**DOI:** 10.1590/2317-1782/e20240237pt

**Authors:** Monique Ramos Paschoal Dutra, Rodrigo Oliveira da Fonsêca, Aryelly Dayane da Silva Nunes-Araújo, Sheila Andreoli Balen

**Affiliations:** 1 Maternidade Escola Januário Cicco – MEJC, Empresa Brasileira de Serviços Hospitalares – EBSERH - Natal (RN), Brasil.; 2 Laboratório de Inovação Tecnológica em Saúde – LAIS, Universidade Federal do Rio Grande do Norte – UFRN - Natal (RN), Brasil.; 3 Departamento de Fonoaudiologia, Universidade Federal do Rio Grande do Norte – UFRN - Natal (RN), Brasil.

**Keywords:** Audição, Perda Auditiva, Políticas Públicas de Saúde, Sistema Único de Saúde, Sistemas de Informação em Saúde

## Abstract

**Objetivo:**

Analisar a produção ambulatorial do visual reinforcement audiometry (VRA) no Sistema Único de Saúde (SUS), nas Unidades da Federação (UFs) do Brasil, entre 2004 e 2021, e sua associação com a Triagem Auditiva Neonatal (TAN) e a oferta de fonoaudiólogos, serviços de saúde auditiva e equipamentos de VRA.

**Método:**

Estudo ecológico misto, que utilizou dados do Sistema de Informações Ambulatoriais do SUS, do Cadastro Nacional de Estabelecimentos de Saúde e do Instituto Brasileiro de Geografia e Estatística. A variável dependente foi a taxa da realização do procedimento “audiometria de reforço visual (via aérea/óssea)”, por 10 mil habitantes, enquanto as variáveis independentes foram as taxas de TAN, fonoaudiólogos, serviços de saúde auditiva e equipamentos de VRA. Foi realizada estatística descritiva e inferencial e análise espacial.

**Resultados:**

A taxa de realização de VRA no Brasil foi de 11,8/10 mil habitantes entre 2004 e 2021. Nas UFs, as taxas de VRA variaram de 0 a 125,1/10 mil habitantes nos períodos estudados. A menor taxa deste exame foi registrada no triênio 2004-2006, ao passo que a maior foi visualizada no triênio 2007-2009. Constatou-se que não houve correlação significativa entre a taxa de realização de VRA do triênio 2016-2018 com as taxas de TAN, fonoaudiólogos, serviços de saúde auditiva e equipamentos de VRA.

**Conclusão:**

A realização do VRA é restrita e discrepante entre as UFs brasileiras e não apresenta associação com as variáveis investigadas, apontando a necessidade de medidas para melhorar o acesso a este exame no SUS.

## INTRODUÇÃO

A Organização Mundial de Saúde estima que, até 2050, cerca de 2,5 bilhões de pessoas apresentarão algum grau de perda auditiva^(1)^. Na infância, a perda auditiva pode ocasionar prejuízos substanciais ao comprometer o desenvolvimento da linguagem, a comunicação, a aprendizagem e a sociabilidade, o que demanda a realização do diagnóstico e da intervenção em tempo oportuno^(1,2)^, conforme preconizado^(3)^.

O processo da identificação à intervenção auditiva de uma criança deve ocorrer até os seis meses de vida^(2)^, sendo assegurado no Brasil pelo Sistema Único de Saúde (SUS), que proporciona o acesso da população a diferentes serviços de saúde^(4)^. Historicamente, o SUS foi marcado por avanços que promoveram melhorias para a saúde dos brasileiros^(5)^. Na atenção à saúde auditiva, um dos marcos correspondeu à instituição da Política Nacional de Atenção à Saúde Auditiva (PNASA), pela Portaria GM/MS nº 2.073, de 2004, objetivando a ampliação na cobertura de atendimento às pessoas com deficiência auditiva, por meio de uma rede de cuidados articulada e integrada entre os níveis de atenção do SUS^(6)^. Em 2010, outro importante avanço consistiu na publicação da Lei Federal nº 12.303, que tratou da obrigatoriedade da realização do exame de Emissões Otoacústicas Evocadas (EOAE) em todas as crianças nascidas em hospitais e maternidades^(7)^.

A PNASA foi revogada em 2011, logo após a instituição do Plano Nacional dos Direitos da Pessoa com Deficiência – Plano Viver sem Limite, por meio do Decreto nº 7.612, que articulou políticas, programas e ações, com vistas a melhorar o acesso à educação, à atenção à saúde, à inclusão social e à acessibilidade no país^(8)^. No ano seguinte, foi criada a Rede de Cuidados à Pessoa com Deficiência (RCPD), pela Portaria GM/MS nº 793, visando a ampliação do acesso e a qualificação do atendimento às pessoas com deficiência^(9)^.

Em paralelo aos avanços da legislação brasileira na atenção à saúde auditiva, é sabido que, para a efetividade do diagnóstico e da intervenção, tornam-se primordiais fonoaudiólogos e equipamentos especializados, à luz de protocolos baseados em evidências científicas, a fim de determinar o tipo e o grau da perda auditiva, para, posteriormente, nortear a intervenção necessária^(3)^. Partindo dessa perspectiva, o diagnóstico audiológico infantil vem sendo aprimorado na identificação da perda auditiva em crianças^(10)^, sendo utilizado o princípio do *cross-check* ^(11)^, que consiste no uso de uma bateria de testes para o fechamento e a caracterização do tipo, do grau e da configuração da perda auditiva. Assim, as recomendações em audiologia pediátrica embasam que tanto as avaliações comportamentais quanto eletrofisiológicas e eletroacústicas devem ser incorporadas em uma avaliação completa para confirmar os resultados em vários procedimentos^(12)^.

O diagnóstico audiológico que ocorre nos Centros Especializados de Reabilitação ou nos Serviços de Saúde Auditiva no Brasil deve seguir recomendações de evidências científicas. Nesse sentido, entre o nascimento até os 5/6 meses de idade, o diagnóstico audiológico prioriza procedimentos eletrofisiológicos e eletroacústicos associados a observações do comportamento auditivo, em virtude dos processos neuromaturacionais da criança nesse período. A avaliação comportamental ou psicoacústica da audição é considerada o padrão-ouro para estimativa dos níveis mínimos da audição, de modo que, na faixa etária de 5/6 até os 24 meses, o procedimento recomendado é a Audiometria de Reforço Visual (*visual reinforcement audiometry –* VRA)^(3)^.

O VRA tem como base uma resposta condicionada (virar a cabeça no plano horizontal) em resposta ao tom puro e aos estímulos de fala, utilizando estímulo visual para o reforço da resposta^(13)^. Quando o VRA é conduzido de acordo com protocolos cuidadosos de estímulo, resposta e condicionamento, ​​podem ser obtidos resultados válidos e confiáveis^(3)^, que têm correlação com os limiares auditivos obtidos mais tardiamente na audiometria lúdica^(14)^. Ademais, os resultados do VRA sugerem uma boa correlação dos níveis mínimos de resposta no VRA com os limiares eletrofisiológicos, por meio dos exames de Potenciais Evocados Auditivos de Tronco Encefálico por Frequência Específica^(10,15)^.

As Diretrizes de Atenção da Triagem Auditiva Neonatal (TAN) no Brasil orientam a realização da avaliação audiológica com o exame de VRA com fones de inserção e medidas de imitância acústica nas etapas de diagnóstico audiológico (falha na TAN) e de monitoramento auditivo (passa na TAN com Indicador de Risco para Deficiência Auditiva)^(16)^. Em adição, o VRA é indicado na avaliação de crianças com mais de 24 meses que sinalizam atraso no desenvolvimento ou interesses restritos, como no Transtorno do Espectro Autista^(13)^.

Ao longo dos anos, estudos têm averiguado o declínio de registros do VRA no Brasil e merece nota, também, que o conhecimento acerca da evolução deste exame, no âmbito inter-regional e intrarregional, ainda é limitado^(17,18)^. Acresce-se a isso o cenário das profundas discrepâncias na atenção à saúde auditiva que ainda se sucedem no país^(19,20)^, a exemplo da distribuição desigual dos serviços de saúde auditiva^(17)^.

Em outro plano, há de se reconhecer que houve relevante incremento da oferta de fonoaudiólogos no SUS^(21)^ e de equipamentos de TAN^(19)^. Ante essas questões, é necessário compreender a realização do VRA frente à sua interface com tais fatores, vislumbrando o apoio à tomada de decisão em saúde, à otimização de recursos públicos e à consonância com evidências científicas. Recentemente, a British Society of Audiology publicou recomendações atualizadas sobre as boas práticas na realização do VRA junto ao diagnóstico audiológico infantil^(22)^.

Face ao exposto, o objetivo deste estudo foi analisar a produção ambulatorial do VRA no SUS, nas Unidades da Federação (UFs) do Brasil, entre 2004 e 2021, e sua associação com a TAN e a oferta de fonoaudiólogos, serviços de saúde auditiva e equipamentos de VRA.

## MÉTODO

Trata-se de um estudo ecológico misto, cujas unidades de análise foram as UFs do Brasil. Para o levantamento de dados, utilizou-se como fontes o Sistema de Informações Ambulatoriais do SUS (SIA/SUS), o Cadastro Nacional de Estabelecimentos de Saúde (CNES) e o Instituto Brasileiro de Geografia e Estatística (IBGE). A coleta de dados ocorreu durante o mês de julho de 2022.

O recorte temporal investigado compreendeu o período de janeiro de 2004, dada a implantação da PNASA neste ano, até dezembro de 2021. Para fins de análise, o período foi segmentado em seis intervalos, perfazendo um total de 18 anos, a saber: 2004-2006; 2007-2009; 2010-2012; 2013-2015; 2016-2018; e 2019-2021, referentes às taxas 1, 2, 3, 4, 5 e 6, respectivamente.

O acesso aos dados ocorreu na plataforma do Departamento de Informática do SUS – DATASUS, do Ministério da Saúde, por meio dos itens “informações de saúde (TABNET)”, “assistência à saúde” e “local de atendimento”. Subsequentemente, foram selecionados os exames “audiometria de reforço visual (via aérea/óssea)” e “emissões otoacústicas evocadas para triagem auditiva”. O procedimento escolhido para representar a TAN foi o exame de EOAE, em razão de ser o mais utilizado na literatura científica. A extração de dados relacionados ao número de fonoaudiólogos foi sequenciada por “rede assistencial”, “CNES – recursos humanos” e “profissionais”.

Ainda no DATASUS, para localizar os quantitativos de serviços de saúde auditiva e equipamentos de VRA no SUS, considerou-se as UFs e a competência “dezembro de cada ano”. Para os serviços de saúde auditiva, selecionou-se “relatórios – serviços especializados – serviço de atenção à saúde auditiva” e, na “consulta de estabelecimentos”, foi especificado o campo “centro especializado em reabilitação”. Quanto aos equipamentos de VRA, a coleta deu-se pelo acesso a “relatórios – equipamentos” e “tipo de equipamento – equipamentos de audiologia – sistema completo de reforço visual (VRA)”. As estimativas populacionais utilizadas foram derivadas do IBGE, que disponibiliza projeções anuais por UFs. Este estudo compreendeu, pela constante de 10.000, a população entre 0 e 4 anos de idade para o cálculo das taxas.

A variável dependente foi descrita pela taxa da realização do procedimento “audiometria de reforço visual (via aérea/óssea)”, por 10 mil habitantes, entre 0 e 4 anos de idade, enquanto as variáveis independentes foram as taxas de TAN, fonoaudiólogos, serviços de saúde auditiva e equipamentos de VRA, coletadas somente para o penúltimo triênio, uma vez que, no último triênio, ocorreu a pandemia de covid-19. Para todas variáveis, foram calculadas taxas que envolveram médias do quantitativo da variável para o triênio e da população estimada, segundo ano do triênio, conforme ilustrado no Quadro 1.

**Quadro 1:** Apresentação das variáveis do estudo com a definição e a fórmula dos cálculos utilizados

Variável	Fórmula
Taxa de realização da Audiometria de Reforço Visual	Média do número de procedimentos de Audiometria de Reforço Visual no triênio/ população com idade entre 0 e 4 anos no segundo ano do triênio x 10 mil habitantes.
Taxa de realização da Triagem Auditiva Neonatal	Média do número de procedimentos “Emissões Otoacústicas Evocadas para Triagem Auditiva” no triênio/ população com idade entre 0 e 4 anos no segundo ano do triênio x 10 mil habitantes.
Taxa de fonoaudiólogos	Média do número de fonoaudiólogos atuantes no Sistema Único de Saúde no triênio/ população com idade entre 0 e 4 anos no segundo ano do triênio x 10 mil habitantes.
Taxa de serviços de saúde auditiva	Média do número de serviços de saúde auditiva e centro especializado em reabilitação no Sistema Único de Saúde no triênio/ população com idade entre 0 e 4 anos no segundo ano do triênio x 10 mil habitantes.
Taxa de equipamentos de Audiometria de Reforço Visual	Média do número de equipamentos de Audiometria de Reforço Visual disponíveis no Sistema Único de Saúde no triênio/ população com idade entre 0 e 4 anos no segundo ano do triênio x 10 mil habitantes.

Os dados foram compilados e organizados em um banco de dados no programa estatístico *Statistical Package for the Social Sciences*, versão 18.0, no qual foi realizada estatística descritiva e inferencial. Inicialmente, foi testada a normalidade da distribuição da variável dependente por meio do teste de Shapiro-Wilk, com α = 5%. Como a distribuição não foi normal, utilizou-se as medidas de tendência central e dispersão e o teste de Spearman para investigar as correlações entre as variáveis dependente e independentes, também com α = 5%. Em seguida, por meio do programa TabWin, versão 4.15, efetuou-se a análise exploratória das taxas de realização do procedimento “audiometria de reforço visual (via aérea/óssea)”, por 10 mil habitantes, entre 0 e 4 anos de idade, nos seis períodos examinados, utilizando-se a distribuição por quartis. Nos mapas, a intensidade dos níveis de cinza está relacionada à magnitude das taxas, ou seja, quanto mais escuro, mais altos são os valores.

O presente estudo apresentou dispensa do Comitê de Ética em Pesquisa, em virtude do delineamento ecológico e da coleta de dados em banco de domínio público, estando em conformidade com a Resolução nº 510/2016 do Conselho Nacional de Saúde^(23)^.

## RESULTADOS

No período investigado, constatou-se que a taxa de realização de VRA no Brasil apresentou oscilações entre 1,6 e 19,1 para cada 10 mil habitantes, entre 0 e 4 anos de idade. A menor taxa foi registrada no período de 2004 a 2006 e a maior no período seguinte, de 2007 a 2009 (Figura 1).

**Figura 1:** Taxa de realização de VRA para cada 10 mil habitantes, entre 0 e 4 anos de idade, no período de 2004 a 2021 no Brasil

**Legenda:** VRA = *visual reinforcement audiometry*

Houve destaque para a região Nordeste, pois a Paraíba apresentou as melhores taxas nos mapas de todos os triênios analisados e o Maranhão esteve dentre as UFs com os maiores valores das taxas a partir do mapa do triênio 2007-2009. As unidades de Rondônia e do Distrito Federal também foram evidenciadas. O mapa do triênio 2004-2006 apresentou as menores taxas, oscilando entre 0 e 8,7 para cada 10 mil habitantes, entre 0 e 4 anos de idade, sob uma maior variedade entre as unidades, ao passo que o mapa do triênio 2007-2009 manifestou as maiores taxas, variando de 31,3 a 125,1 (Figura 2).

**Figura 2:** Distribuição espacial da taxa de realização de VRA para cada 10.000 habitantes, entre 0 e 4 anos de idade, no período de 2004 a 2021 por Unidade da Federação do Brasil

De acordo com a Tabela 1, a taxa de TAN do Brasil foi de 1.483,20/10.000 habitantes, entre 0 e 4 anos de idade, e variou entre 581,3, no Mato Grosso, e 3.324,8, no Amapá (média = 1.729,6; DP = 1.387,8- 2.071,4). Em relação à taxa de fonoaudiólogos, a taxa do Brasil foi de 13,70/10.000 habitantes, entre 0 e 4 anos de idade, sendo que Minas Gerais apresentou a maior taxa, 18,90, e o Amazonas obteve a menor, 4,4 (média = 11,3; DP = 9,7-12,9). Quanto aos serviços de saúde auditiva, a taxa do Brasil foi de 0,4/10.000 habitantes, entre 0 e 4 anos de idade, com variação entre 0,1, em Alagoas, e 1,2, no Paraná (Md = 0,3; IIQ = 0,2). Por fim, a taxa de equipamentos de VRA do Brasil foi de 0,13/10.000 habitantes, entre 0 e 4 anos de idade, na qual a maior taxa foi registrada em Goiás, no valor de 0,6, e as unidades de Rondônia, Acre, Amapá, Tocantins, Maranhão e Sergipe apresentaram taxas com valores nulos (Md = 0,1; IIQ = 0,2). A Tabela 1 considerou a sequência padronizada do IBGE no tocante ao ordenamento das Unidades da Federação, tendo início por Rondônia e término pelo Distrito Federal.

**Tabela 1:** Taxas de realização de VRA, TAN, fonoaudiólogos, serviços de saúde auditiva e equipamentos de VRA para cada 10.000 habitantes, entre 0 e 4 anos de idade, no período de 2016 a 2018 por Unidade da Federação do Brasil

UF	TXVRA	TXTAN	TXFONO	TXSS	TXEQUIP
Rondônia	26,80	891,40	9,40	0,4	0,00
Acre	0,00	2.018,60	5,00	0,4	0,00
Amazonas	0,20	1.216,30	4,40	0,2	0,10
Roraima	0,00	2.746,80	7,50	0,2	0,10
Pará	16,20	1.089,70	5,60	0,3	0,10
Amapá	0,00	3.324,80	8,80	0,5	0,00
Tocantins	0,30	1.451,90	9,40	0,8	0,00
Maranhão	39,90	786,50	6,50	0,3	0,00
Piauí	0,10	2.530,90	12,90	0,2	0,20
Ceará	9,30	1.217,70	9,70	0,2	0,10
Rio Grande do Norte	22,80	1.611,30	13,10	0,6	0,40
Paraíba	106,90	2.558,60	13,30	0,3	0,10
Pernambuco	0,70	827,20	10,70	0,4	0,10
Alagoas	4,10	1.263,10	9,30	0,1	0,10
Sergipe	0,00	1.630,00	9,50	0,3	0,00
Bahia	17,80	1.003,90	8,20	0,3	0,10
Minas Gerais	4,00	1.261,30	18,90	0,3	0,10
Espírito Santo	8,30	1.417,00	13,00	0,3	0,20
Rio de Janeiro	5,60	1.036,30	17,20	0,4	0,10
São Paulo	21,50	1.167,90	18,70	0,5	0,20
Paraná	12,70	3.306,90	16,90	1,2	0,20
Santa Catarina	1,90	2.010,30	17,20	0,6	0,20
Rio Grande do Sul	6,50	3.259,50	15,20	0,6	0,10
Mato Grosso do Sul	12,50	3.055,60	13,30	0,5	0,30
Mato Grosso	2,80	581,30	8,80	0,2	0,20
Goiás	23,20	963,80	12,10	0,5	0,60
Distrito Federal	30,70	2.471,70	12,20	0,3	0,00
Total	14,70	1.483,20	13,70	0,4	0,13

**Legenda:** VRA = *visual reinforcement audiometry*; UF = Unidade da Federação; TXVRA= taxa de realização de VRA; TXTAN = taxa de Triagem Auditiva Neonatal; TXFONO = taxa de fonoaudiólogos; TXSS = taxa de serviços de saúde auditiva; TXEQUIP = taxa de equipamentos de VRA

Na análise estatística inferencial, observou-se que não houve correlação significativa entre a taxa de realização de VRA do penúltimo triênio com as taxas de TAN, fonoaudiólogos, serviços de saúde auditiva e equipamentos de VRA. A taxa de fonoaudiólogos apresentou correlação estatisticamente significativa com as taxas de serviços de saúde auditiva e equipamentos de VRA (Tabela 2).

**Tabela 2:** Análise da correlação entre a taxas de VRA, TAN, fonoaudiólogos, equipamentos de VRA e serviços de saúde auditiva para cada 10.000 habitantes, entre 0 e 4 anos de idade, no período de 2016 a 2018 no Brasil

Variáveis	TX VRA	TX TAN	TX FONO	TX EQUIP	TX SS
TX VRA	1,000				
TX TAN	-0,269	1,000			
TX FONO	0,241	0,257	1,000		
TX EQUIP	0,191	0,015	0,486^*^	1,000	
TX SS	0,138	0,274	0,464*	0,369	1,000

**Legenda:** VRA = *visual reinforcement audiometry*; TXTAN = taxa de Triagem Auditiva Neonatal; TXFONO = taxa de fonoaudiólogos; TXSS = taxa de serviços de saúde auditiva; TXEQUIP = taxa de equipamentos de VRA

* correlação moderada

## DISCUSSÃO

Os resultados do presente estudo permitiram identificar a variabilidade da realização do VRA no SUS no decorrer dos anos. As UFs analisadas exibiram, simultaneamente, importantes disparidades no que concerne às taxas de TAN, fonoaudiólogos, serviços de saúde auditiva e equipamentos de VRA. Este contexto é considerado preocupante, visto que este exame, imprescindível para o diagnóstico audiológico infantil na faixa etária de 5/6 a 24 meses, perpassou por decréscimos temporais importantes.

As maiores taxas da realização do VRA no Brasil, sinalizadas no intervalo de 2007 a 2009, alinham-se aos progressos que a PNASA propiciou para a atenção à saúde auditiva^(24,25)^, tanto que, com a sua instituição, o acesso aos cuidados na área foi ampliado progressivamente, refletindo no aumento da execução de procedimentos audiológicos^(17)^. Sob essa premissa, as menores taxas da realização do VRA, registradas entre 2004 e 2006, estão atreladas ao processo ainda recente de implantação da PNASA no país, o que permite compreender tais achados. É necessário ressaltar, também, que a PNASA não apresentou recomendações específicas para a realização do VRA no SUS^(6)^.

Dada a evolução geral verificada neste estudo, convém salientar o comportamento das taxas da realização do VRA no SUS nos demais triênios analisados, uma vez que, de 2010 a 2012, houve uma intensa diminuição quando comparado o ápice registrado entre 2007 e 2009, seguida pela discreta variação positiva de 2013 a 2015 e por quedas nos triênios posteriores. Os resultados corroboram o estudo realizado em Salvador - BA, que apontou uma tendência de crescimento geral da produção ambulatorial em saúde auditiva, sobretudo com pico no ano de 2007, e variações nos outros anos averiguados^(26)^. O panorama investigado reforça os resultados de uma pesquisa que detectou aumento das taxas de cobertura da TAN, entre 2008 e 2015, no Brasil^(27)^.

À vista disso, é válido ponderar as modificações junto às políticas que envolveram a saúde auditiva no SUS, como a revogação da PNASA, a publicação da Lei Federal nº 12.303, a implantação do Plano Viver sem Limite e a criação da RCPD. Em meio à transição de tais medidas, ocorreu o crescimento da realização de procedimentos audiológicos no SUS, ainda que de forma desigual entre as regiões brasileiras e os tipos de procedimentos efetuados, a exemplo da diminuição de 28,26% na execução do VRA no SUS^(18)^.

De acordo com os achados da realização do VRA no SUS nos triênios analisados, verificou-se que as maiores taxas derivaram da Paraíba, do Maranhão, de Rondônia e do Distrito Federal. A Paraíba, que figurou nos mapas de todos os triênios com as taxas mais proeminentes do procedimento, já despontou na literatura em função de realçar, também, as melhores coberturas de TAN do Brasil^(27)^ e de fonoaudiólogos no SUS^(19)^. Na contramão desses resultados, o comportamento das taxas de TAN e de fonoaudiólogos no SUS, no período de 2016 a 2019, mostrou-se distinto, com maiores amplitudes no Amapá e em Minas Gerais, respectivamente.

A distribuição das taxas de TAN com valores mais elevados quando contrastadas às de VRA decorrem das políticas públicas e da necessidade da sua realização em todos os nascidos vivos do Brasil^(16)^. A correlação negativa da taxa de realização de VRA com a taxa de TAN revela uma lacuna no programa de TAN Universal, considerando que os locais onde a triagem tem maior execução estão realizando menos exames de VRA, o que ilustra a ocorrência de evasão nas etapas de monitoramento e diagnóstico^(28)^. Por outro lado, vale pontuar que o sistema de informação em saúde empregado não especifica a idade de conclusão do diagnóstico audiológico, que, por sua vez, está propenso a um padrão de variabilidade frente a diversos aspectos inerentes às UFs brasileiras.

A distribuição das taxas de fonoaudiólogos no SUS nas regiões Sul e Sudeste condiz com o histórico dos cursos de graduação em Fonoaudiologia no Brasil e com os indicadores sociais^(21)^, haja vista que as maiores taxas da oferta de fonoaudiólogos foram localizadas em UFs destas regiões, onde há predomínio de especialistas em audiologia e de programas de formação na área, elementos indispensáveis na efetividade da atenção prestada^(24)^. Tão logo, a execução do VRA depende da experiência e do treinamento específico de audiologistas durante o seu processo de realização, baseando-se em evidências científicas^(13,22)^. Vale ressaltar que o fonoaudiólogo possui outras áreas de atuação no SUS, além da audiologia e sua atuação é cada vez mais necessária no âmbito das equipes multiprofissionais em todos os níveis de complexidade.

Adicionalmente, um dos desafios potenciais que se identifica no processo de realização desse procedimento refere-se à disponibilidade de recursos nos serviços de saúde auditiva^(29)^. Dentro dessa perspectiva e pautando-se nos achados deste estudo, as UFs demonstraram limitada presença de equipamentos de VRA, dado que algumas UFs, especialmente das regiões Norte e Nordeste, atestaram taxas nulas. Tais achados revelam a importância de investimentos em recursos físicos no SUS^(4,19)^, tendo em vista que o equipamento para a realização do VRA envolve um sistema específico com cabina maior e materiais específicos com brinquedos luminosos^(22)^.

É de conhecimento que os serviços fonoaudiológicos necessitam de investimentos públicos para sua manutenção, sobretudo na área de Audiologia, que engloba procedimentos de maior custo. Nesse aspecto, estudiosos, ao examinarem os gastos públicos voltados aos serviços ambulatoriais em Fonoaudiologia, concluíram que a Audiologia concentrou 95,4% dos recursos destinados aos serviços, com crescimento gradativo de investimentos até 2011 e instabilidades nos anos seguintes. Dentre as regiões, as maiores parcelas de gastos foram registradas nas regiões Sudeste, Nordeste, Sul, Centro-Oeste e Norte, respectivamente, sob notáveis desigualdades^(25)^.

Ao reportar às desigualdades regionais, o presente estudo integra-se ao contexto das marcantes discrepâncias brasileiras para a efetividade da atenção à saúde e do acesso aos serviços^(4)^. Apesar de serem maciças, as desigualdades regionais passaram a ser atenuadas com medidas que ocorreram no SUS para melhorar esse quadro^(5)^. Na atenção à saúde auditiva, houve, entre 2008 e 2012, expansão do credenciamento de novos serviços de saúde auditiva, especialmente nas regiões Norte, Nordeste e Centro-Oeste, bem como de procedimentos audiológicos no SUS, que repercutem diretamente na produção ambulatorial^(30)^.

Ainda assim, conforme os resultados deste estudo, foi possível constatar desigualdades nas taxas de serviços de saúde auditiva, que foram mais baixas em UFs das regiões Norte e Nordeste e mais elevadas em UFs da região Sul, na qual, em 2010, a cobertura estimada de serviços já havia se tornado a maior do país^(17)^. Compreende-se, então, que as lacunas no credenciamento desses serviços são passíveis de marcar ainda mais as desigualdades regionais existentes, implicando no acesso dos usuários aos cuidados em saúde auditiva.

Os achados do presente estudo podem ser justificados pela carência da formação de fonoaudiólogos na área de audiologia infantil, o que expressa a necessidade de maior incorporação da temática desde a formação acadêmica, e pelo fato de haver uma concentração da realização de outros procedimentos audiológicos^(17,18)^, abrindo espaço para debates que envolvam estratégias mais efetivas para melhorar as etapas do diagnóstico^(28)^. Além disso, cabe atenção que o VRA requer a presença de dois avaliadores, diferentemente de outros procedimentos audiológicos, e de uma cabina específica, com dimensões maiores^(22)^. No cerne dessas especificidades, vale destacar que, diante dos avanços tecnológicos e com a maior aquisição de equipamentos para efetuar exames mais objetivos, a realização do VRA no SUS passou a ser menos frequente^(17)^.

Como limitações deste estudo, considera-se o uso de dados dos sistemas de informação em saúde, que podem apresentar dados faltantes ou subnotificados, tampouco sem mecanismos de mensuração dessas dificuldades. Além disso, os dados incluídos referentes a habitantes entre 0 e 4 anos, por padrão das fontes utilizadas, também configuram uma limitação dos sistemas de informação em saúde. Não há referências sobre as taxas da realização do VRA, em função de não haver estudos nacionais com valores de prevalência da deficiência auditiva no público infantil, o que limita uma análise aprofundada das taxas, como também para as taxas de falha na TAN.

A não inclusão do Potencial Evocado Auditivo de Tronco Encefálico como um exame de TAN e os cálculos das taxas, que não excluíram a população com acesso à assistência suplementar, também podem ser mencionados como limitações. O emprego da unidade de análise por UF apresenta valores que podem ser diferentes de acordo com a unidade de análise utilizada, observando-se, para tanto, as particularidades existentes entre os municípios.

A despeito disso, o uso de dados dos sistemas de informação em saúde auxilia no reconhecimento da realidade da saúde auditiva no SUS e na identificação das UFs que necessitam de maior atenção, contribuindo com avaliações para planejamento, implantação e aprimoramento de serviços e ações em saúde pública.

Diante da relevância do diagnóstico audiológico em tempo oportuno e assertivo para o público infantil, organizações científicas têm recomendado, por meio de indicadores, a avaliação dos serviços de saúde e o monitoramento da qualidade^(3,16,22)^. Para o Brasil, sugere-se a inclusão de aspectos relacionados à aquisição e à manutenção de recursos humanos, físicos e materiais nas políticas públicas, bem como a realização de estudos com análises de procedimentos audiológicos direcionados à intervenção na deficiência auditiva, como o aparelho de amplificação sonora individual e o implante coclear.

## CONCLUSÃO

A realização do VRA apresenta decréscimos importantes ao longo dos anos no Brasil e discrepâncias na sua distribuição entre as UFs, reportando para a necessidade de medidas capazes de minimizar o impacto da restrição deste exame no SUS. A ausência de correlação entre as taxas de VRA e TAN retratam a descontinuidade dos programas de TAN, isto é, as UFs que apresentam altas coberturas de TAN não estão demonstrando elevados valores para o exame do VRA, bem como as UFs que possuem maior número de fonoaudiólogos, serviços de saúde auditiva e equipamentos de VRA estão realizando o exame em menor quantidade.

Diante dos achados, é possível inferir que as disparidades para a realização do VRA no SUS impactam negativamente a atenção à saúde auditiva, a ponto de que crianças pequenas podem estar sem acesso a este exame no processo de avaliação audiológica.
